# Cognitive Dysfunction Is Sustained after Rescue Therapy in Experimental Cerebral Malaria, and Is Reduced by Additive Antioxidant Therapy

**DOI:** 10.1371/journal.ppat.1000963

**Published:** 2010-06-24

**Authors:** Patricia A. Reis, Clarissa M. Comim, Fernanda Hermani, Bruno Silva, Tatiana Barichello, Aline C. Portella, Flavia C. A. Gomes, Ive M. Sab, Valber S. Frutuoso, Marcus F. Oliveira, Patricia T. Bozza, Fernando A. Bozza, Felipe Dal-Pizzol, Guy A. Zimmerman, João Quevedo, Hugo C. Castro-Faria-Neto

**Affiliations:** 1 Laboratório de Imunofarmacologia, Instituto Oswaldo Cruz, Fundação Oswaldo Cruz, Rio de Janeiro, Brazil; 2 Laboratório de Neurociências and Instituto Nacional de Ciência e Tecnologia Translacional em Medicina, Universidade do Extremo Sul Catarinense, Criciúma, Brazil; 3 Instituto de Ciências Biomédicas Universidade Federal do Rio de Janeiro, Rio de Janeiro, Brazil; 4 Instituto de Bioquímica Médica, Universidade Federal do Rio de Janeiro, Rio de Janeiro, Brazil; 5 Instituto de Pesquisa Clínicas Evandro Chagas, Fundação Oswaldo Cruz, Rio de Janeiro, Brazil; 6 Laboratório de Fisiopatologia Experimental, Universidade do Extremo Sul Catarinense, Criciúma, Brazil; 7 Department of Medicine and Program in Human Molecular Biology and Genetics, University of Utah, Salt Lake City, Utah, United States of America; National Institute for Medical Research, United Kingdom

## Abstract

Neurological impairments are frequently detected in children surviving cerebral malaria (CM), the most severe neurological complication of infection with *Plasmodium falciparum*. The pathophysiology and therapy of long lasting cognitive deficits in malaria patients after treatment of the parasitic disease is a critical area of investigation. In the present study we used several models of experimental malaria with differential features to investigate persistent cognitive damage after rescue treatment. Infection of C57BL/6 and Swiss (SW) mice with *Plasmodium berghei* ANKA (PbA) or a lethal strain of *Plasmodium yoelii* XL (PyXL), respectively, resulted in documented CM and sustained persistent cognitive damage detected by a battery of behavioral tests after cure of the acute parasitic disease with chloroquine therapy. Strikingly, cognitive impairment was still present 30 days after the initial infection. In contrast, BALB/c mice infected with PbA, C57BL6 infected with *Plasmodium chabaudi chabaudi* and SW infected with non lethal *Plasmodium yoelii NXL* (PyNXL) did not develop signs of CM, were cured of the acute parasitic infection by chloroquine, and showed no persistent cognitive impairment. Reactive oxygen species have been reported to mediate neurological injury in CM. Increased production of malondialdehyde (MDA) and conjugated dienes was detected in the brains of PbA-infected C57BL/6 mice with CM, indicating high oxidative stress. Treatment of PbA-infected C57BL/6 mice with additive antioxidants together with chloroquine at the first signs of CM prevented the development of persistent cognitive damage. These studies provide new insights into the natural history of cognitive dysfunction after rescue therapy for CM that may have clinical relevance, and may also be relevant to cerebral sequelae of sepsis and other disorders.

## Author Summary

Cerebral malaria (CM) is a deadly consequence of *Plasmodium falciparum* infection. Severe neurologic deficits are frequent during CM. Although most resolve within 6 months, several retrospective studies have described high frequencies of long-lasting cognitive impairment after an episode of CM. We developed behavioral tests to identify cognitive impairment due to experimental CM. During infection with *Plasmodium berghei* ANKA (PbA), mice susceptible to CM (C57BL/6) developed long-lasting cognitive impairment in contextual and aversive memory. The same profile was seen in Swiss Webster mice infected with *Plasmodium yoelii* XL, a lethal strain that also induces neurological dysfunctions in susceptible mice strains, confirming that the cognitive dysfunction is closely associated to the development of CM. Reactive oxygen species are described as mediators of neurological and cognitive impairment associated to sepsis and Alzheimer's disease. Here we found enhanced production of malondialdeyde and conjugated dienes in brains of PbA-infected C57BL/6 mice, indicating oxidative stress. Antioxidant therapy with N-acetylcisteine and desferroxamine, as an additive to chloroquine, prevented the cognitive impairment, confirming the importance of oxidative stress in CM-associated cognitive sequellae. Administration of additive antioxidants may be a successful therapeutic strategy to control long-lasting consequences of CM and in other severe systemic inflammatory syndromes with neurological involvement.

## Introduction

Malaria, together with tuberculosis and human immunodeficiency virus/acquired immunodeficiency syndrome (HIV/AIDS), is one of three most important infectious diseases worldwide, with devastating morbidity and mortality and deleterious economic consequences [1]. More than 400 million people suffer from malaria, which causes over two million deaths annually, mainly among African children [2]. Cerebral malaria (CM) is the most severe neurological complication of infection with *Plasmodium falciparum* and is the main cause of acute non-traumatic encephalopathy in tropical countries. Mortality is high. In addition, physical and neurologic deficits are frequently seen at the time of hospital discharge in children surviving CM, although most resolve within 6 months after discharge [3]. Nevertheless, several retrospective studies suggest that cognitive deficits in children with CM are more frequent, and persist far longer than physical and neurologic deficits [4], [5], [6], [7], [8]. Boivin et al. [4] reported that 21% of children >5 years old with CM have cognitive deficits 6 months after discharge, and that increased seizure frequency and prolonged coma duration are associated with persistent cognitive deficits. Desruisseaux and coworkers [9] reported cognitive dysfunction in the acute phase of experimental infection with *Plasmodium berghei* ANKA in mice. A test of work memory performed at the 7th day of infection demonstrated significant impairment in visual memory in C57BL/6 mice associated to significant histological alterations as well as hemorrhage and inflammation [9].

Although the pathogenesis of CM has been extensively investigated, many aspects of the cellular and molecular pathogenesis remain incompletely defined [10]. This is in part due to the complexity of the host-pathogen interaction, which includes intricate biologic and inflammatory responses, variations in immune status and genetic background of the host, and factors unique to the malarial parasites [1]. This complexity has been revealed by clinical and experimental observations that have recently included informative mouse models [11], [12], [13]. Biochemical features also influence the natural history and complications of CM [14]. For example, there is evidence that oxidative stress mediates some of the tissue damage caused by experimental malarial infection and in cultured human cells [15], [16].

Until recently physicians have focused on survival of patients with CM and not on long-term outcomes and sequellae and, as a result, the incidence and impact of chronic neurocognitive dysfunction have been underestimated and underreported [17]. Similarly, there has been little inquiry into these issues in experimental CM. Here, we establish and characterize permissive and resistant murine models that clearly demonstrate sustained cognitive dysfunction due to CM. In addition, we demonstrate that a component of the cognitive dysfunction is related to oxidative stress and that this can be favorably modified by an interventional strategy that includes antioxidants in addition to specific rescue chemotherapy aimed at the malarial pathogen. Because oxidative stress is a pathogenetic mechanism in other syndromes of neurocognitive injury and neurodegeneration, the findings may also be relevant to other systemic inflammatory syndromes with cerebral involvement.

## Results

### Characterization of different models of malaria in mice

**Figure 1:**
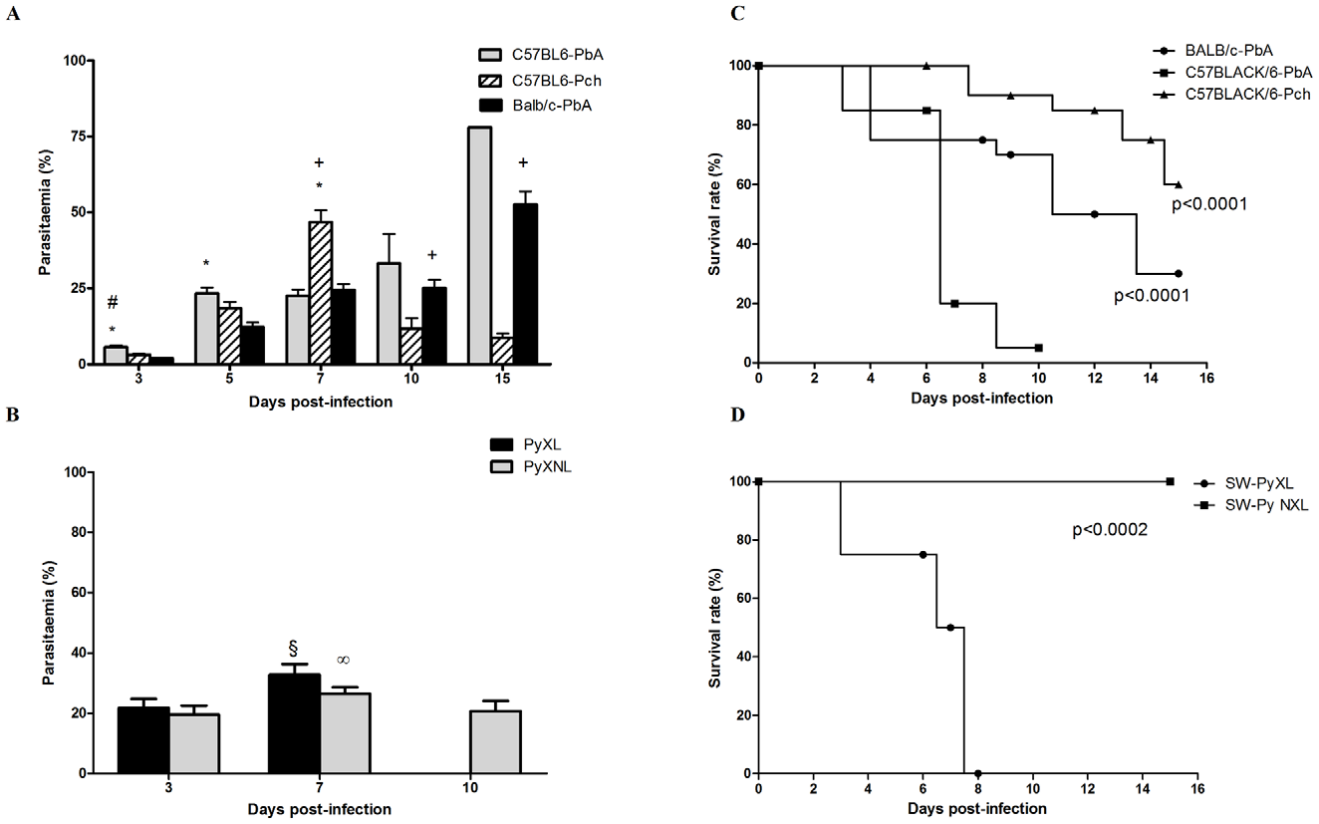
Time-course of parasitemia and survival rate of C57BL6, BALB/c and Swiss Webster (SW) mice after infection with PbA, Pch, PyNXL or PyXL (n = 12–20/group). Date on parasitemia (A and B) are shown as mean ± SEM. Comparisons of C57BL6-PbA versus C57BL6-Pch (#), C57BL6-PbA versus BALB/c-PbA (*) and C57BL6-Pch versus BALB/c-PbA (+), were significant by Tukey's Multiple Comparison Test (A). (θ) and (∞) indicate p<0.05 in relation to day 3 post-infection during PyNXL and PyXL infection, respectively (B). Survival curves (C and D) were evaluated by Log-rank (Mantel-Cox) and Gehan-Breslow-Wilcoxon tests.

In order to establish the clinical course of neurobehavioural complications of CM, mice from diverse genetic backgrounds were infected with different strains of *Plasmodium*. Mortality, parasitemia and behavior alterations (detected by the SHIRPA protocol – see below) were recorded. First, we compared C57BL/6 and BALB/c mice infected with PbA (Figure 1A, C). Ninety five percent of C57BL/6 mice died between 7 and 10 days (with an average of 7.7 days of survival, Figure 1C) after infection, with cerebral manifestations including convulsion, paralysis, and coma. Mean parasitaemia was 23%. Seventy percent of BALB/c mice died within 15 days after infection with severe anemia and overwhelming parasitemia (∼80%), but no signs of cerebral malaria. C57BL/6 mice had 40% mortality within 15 days after infection with Pch (Figure 1C). These animals showed high parasitemia (average of 46%) on day 7 and profound anemia, but no signs of CM were observed at anytime during the experiment. In this model, parasitemia at day 10 post infection was an average of 11% in surviving mice. In an additional model of infection, used to examine the effect of genetic background and parasite variables, SW mice infected with PyXL had 100% lethality (Figure 1D) within 8 days, surviving an average of 7.25 days and displaying clear signs of CM at day 6 associated with substantial parasitemia (approximately 32%). In contrast, when SW mice were infected with the non-lethal PyNXL, 100% of the animals survived for at least 15 days post infection with parasitemia over 20% and no signs of CM. These results are in agreement with others in the literature [12], [13], [18] and confirm that C57BL/6 and SW mice are susceptible to CM when infected with PbA or the lethal strain of Py, respectively.

### Early neurological alterations of CM in susceptible strains are detected by screening with the SHIRPA protocol

The summary results of primary screening by SHIRPA on days 3 and 6 post-infection are shown in Table 1. On day 3 post-infection, no alterations were observed in any of the groups tested (C57BL/6 versus BALB/c infected with PbA; C57BL/6 infected with PbA versus Pch; or SW mice infected with PyNXL versus PyXL) (Table 1). On day 6, however, C57BL/6 mice infected with PbA and SW infected with PyXL displayed significant alterations of reflex and sensory function, motor behavior, and autonomic function. On day 7, the animals demonstrated additional alterations of muscle tone and strength (not shown). BALB/c mice showed minor alterations of motor behavior and autonomic function (only in two tests in this protocol, while susceptible C57BL6 infected with PbA and SW mice infected with PyXL displayed positive findings in three and four assessments, respectively). Previously, Lackner et al. [19] reported that alterations of autonomic function and muscle tone and strength are specific and early signs of CM. Using these criteria, the SHIRPA protocol was prospectively applied on day 6 to identify CM. Positive results diagnosing CM were then taken as an indication to start chloroquine treatment, and to conduct further assessment of cognitive function in CM-positive animals. Interestingly, when we started treatment with chloroquine at 25 mg/kg on day 6, signs of neurological involvement were rapidly responsive and were abolished by day 7 post infection (data not shown).

**Table 1:** Summary of primary screening results.

Tests	C57/BL6						Balb/c				*Swiss webster*					
	RBC		PbA		Pch		RBC		PbA		RBC		PyNXL		Py XL	
	3D	6D	3D	6D	3D	6D	3D	6D	3D	6D	3D	6D	3D	6D	3D	6D
**Reflex and sensory function**																
Visual placing	3 (3/3)	3 (3/3)	3 (3/3)	3 (3/3)	3 (3/3)	3 (3/3)	3 (3/3)	3 (3/3)	3 (3/3)	3 (3/3)	3 (3/3)	3 (3/3)	3 (3/3)	3 (3/3)	3 (3/3)	3 (3/3)
*Pinna* reflex	1 (1/2)	1 (1/2)	1 (1/2)	1 (0/2)	2 (1/2)	1.25 (1/2)	1 (1/2)	1 (1/2)	1 (1/2)	1 (0/2)	1 (1.2/2)	1 (1/1)	1 (1/1)	1 (0/2)	1 (11/1	1 (0/1)
Toe pinch	3 (3/3)	3 (1/3)	3 (1/3)	3 (1/3)	3 (2/3)	3 (1/3)	3 (2/3)	3 (1/3)	3 (3/3)	2 (3/3)	3 (3/3)	3 (1/3)	3 (2/3)	3 (2/3)	3 (0/3)	3 (0/3)
Corneal reflex	1 (1/2)	1 (1/2)	1 (1/2)	1 (0/2)	1 (1/2)	1 (1/2)	2 (1/2)	1.6 (1/2)	1 (1/2)	1 (0/2)	1.4 (1/2)	1.8 (1/2)	1 (1/2)	1.8 (1/2)	2 (1/2)	1 (1/2)
Contact reflex	1 (1/1)	1 (1/1)	1 (1/1)	1 (0/1)	1 (1/1)	1 (1/1)	1 (1/1)	1 (1/1)	1 (1/1)	1 (1/1)	1 (1/1)	1 (1/1)	1 (1/1)	1 (0/1)	1 (1/1)	1 (0/1)
Positional reflex	0 (0/0)	0 (0/0)	0 (0/0)	0 (0/0)	0 (0/0)	0 (0/0)	0 (0/0)	0 (0/0)	0 (0/0)	0 (0/0)	0 (0/0)	0 (0/0)	0 (0/0)	0 (0/0)	0 (0/0)	0 (0/0)
**Neuropsychiatric state**																
*Spontaneous activity*	*2 (2/3)*	*2 (223)*	*2 (2/2)*	***2 (1/2)*** *	*2 (2/2)*	*2 (2/2)*	*2 (2/2)*	*2 (2/2)*	*2 (1/2)*	*2 (1/2)*	*2 (2/2)*	*2 (2/2)*	*2 (2/2)*	*2 (2/2)*	*2 (2/3)*	***1 (0/2)*** *
*Transfer arousal*	*3 (3/5)*	*3 (3/5)*	*3 (3/5)*	***3 (2/5)*** *	*5 (3/5)*	*4 (3/5)*	*3 (3/5)*	*3 (3/5)*	*3 (3/3)*	*3 (2/5)*	*3 (3/3)*	*3 (3/3)*	*3 (3/3)*	*3 (3/4)*	*3 (3/3)*	*3 (1/3)*
Touch escape	3 (2/3)	3 (1/3)	3 (2/3)	3 (1/3)	3 (3/3)	3 (3/3)	3 (2/3)	2 (1/3)	2.5 (2/3)	2 (1/3)	3 (3/3)	3 (3/3)	3 (3/3)	3 (3/3)	3 (3/3)	3 (1/3)
Positional passivity	0 (0/0)	0 (0/0)	0 (0/0)	0 (0/0)	0 (0/0)	0 (0/0)	0 (0/0)	0 (0/0)	0 (0/0)	0 (0/0)	0 (0/0)	0 (0/0)	0 (0/0)	0 (0/0)	0 (0/0)	0 (0/1)
Fear	1 (0/1)	1 (0/1)	1 (0/1)	1 (0/1)	1 (0/1)	0.5 (0/1)	1 (0/1)	1 (0/1)	1 (0/1)	1 (0/1)	1 (1/1)	1 (1/1)	1 (1/1)	1 (1/1)	1 (1/1)	1 (1/1)
Biting	0 (0/0)	0 (0/0)	0 (0/0)	0 (0/0)	0 (0/0)	0 (0/0)	0 (0/0)	0 (0/0)	0 (0/0)	0 (0/0)	0 (0/0)	0 (0/0)	0 (0/0)	0 (0/0)	0 (0/0)	0 (0/0)
Irritability	1 (1/1)	1 (1/1)	1 (1/1)	1 (0/1)	1 (1/1)	1 (1/1)	1 (1/1)	1 (1/1)	1 (1/1)	1 (1/1)	1 (1/1)	1 (1/1)	1 (1/1)	1 (1/1)	1 (1/1)	1 (1/1)
*Vocals*	*1 (1/1)*	*1 (1/1)*	*1 (1/1)*	***1 (0/1)*** *	*1 (1/1)*	*1 (1/1)*	*1 (0/1)*	*1 (0/1)*	*1 (0/1)*	*1 (0/1)*	*1 (0/1)*	*0 (0/1)*	*0 (0/0)*	*1 (0/1)*	*0 (0/0)*	***1 (0/1)*** *
**Motor behavior**																
*Locomotor activity*	*17.6 (5.7)*	*18.0 (6.1)*	*16.3 (4.9)*	***14.2 (4.6)*** *	*17.6 (6.5)*	*21.3 (6.4)*	*16.1 (5.3)*	*19.9 (11.2)*	*11.6 (4.7)*	***6.5 (4.5)*** *	*13.9 (9.0)*	*11.8 (5.5)*	*13.6 (5.2)*	*9.2 (4.6)*	*10.2 (6.0)*	***4.7 (3.8)*** *
Body position	3 (3/3)	3 (3/3)	3 (3/3)	3 (2/3)	3 (3/3)	3 (3/3)	3 (3/3)	3 (3/3)	3 (3/3)	3 (3/3)	3 (3/3)	3 (3/3)	3 (3/3)	3 (3/3)	3 (3/3)	3 (2/3)
*Shivering*	*0 (0/0)*	*0 (0/0)*	*0 (0/0)*	***0 (0/1)*** *	*0 (0/0)*	*0 (0/0)*	*0 (0/0)*	0 (0/0)	*0 (0/0)*	*0 (0/1)*	*0 (0/0)*	*0 (0/0)*	*0 (0/0)*	*0 (0/0)*	*0 (0/0)*	*0 (0/0)*
*Gait*	*0 (0/0)*	*0 (0/0)*	*0 (0/0)*	***1 (0/1)*** *	*0 (0/0)*	*0 (0/0)*	*0 (0/0)*	0 (0/1)	*0 (0/0)*	*0 (0/0)*	*0 (0/0)*	*0 (0/0)*	*0 (0/0)*	*0 (0/1)*	*0 (0/1)*	***0.8 (0/3)*** *
Pelvic elevation	2 (2/2)	2 (2/2)	2 (2/2)	2 (0/3)	2 (2/2)	2 (2/2)	2 (2/2)	2 (2/2)	2 (2/2)	2 (2/2)	2 (1/2)	2 (2/2)	2 (2/2)	2 (2/3)	2 (2/2)	2 (0/3)
*Tail elevation*	*1 (1/2)*	*1 (1/2)*	*1 (1/1)*	***1 (1/2)*** *	*1 (0/2)*	*1 (1/2)*	*1 (1/1)*	*1 (1/1)*	*1 (1/1)*	*1 (1/2)*	*1 (1/1)*	*1 (1/1)*	*1 (1/1)*	*1 (1/2)*	*1 (1/1)*	*1 (0/1)*
Trunk curl	1 (1/1)	1 (1/1)	1 (1/1)	1 (0/1)	1 (1/1)	1 (0/1)	1 (1/1)	1 (1/1)	1 (1/1)	1 (0/1)	1 (1/1)	1 (1/1)	1 (1/1)	1 (1/1)	0 (0/1)	***0 (0/1)*** *
Limb grasping	0 (0/0)	0 (0/1)	0 (0/1)	0 (0/1)	0 (0/0)	0.5 (0/1)	0 (0/0)	0 (0/1)	0 (0/0)	0 (0/1)	0 (0/0)	0 (0/0)	0 (0/0)	0 (0/0)	0 (0/0)	0 (0/1)
*Wire manoeuvre*	*0 (0/1)*	*0 (0/1)*	*1 (0/1)*	***1 (0/3)*** *	*0 (001)*	*0 (0/0)*	*0 (0/1)*	*0 (0/0)*	*0 (0/0)*	*0 (0/1)*	*0 (0/0)*	*0 (0/0)*	*0 (0/1)*	*0.8 (0/1)*	*0 (0/1)*	***1 (0/3)*** *
Negative geotaxis	0 (0/0)	0 (0/0)	0 (0/0)	0 (0/0)	0 (0/0)	0 (0/0)	0 (0/0)	0 (0/0)	0 (0/0)	0 (0/0)	0 (0/0)	0 (0/0)	0 (0/0)	0 (0/0)	0 (0/0)	0 (0/2)
**Autonomous function (early CM parameter)**																
*Respiration rate*	*2 (2/2)*	*2 (2/2)*	*2 (2/2)*	***2 (0/2)*** *	*2 (2/2)*	*2 (2/2)*	*2 (2/2)*	*2 (2/2)*	*2 (0/2)*	***0 (0/2)*** *	*2 (2/2)*	*2 (2/2)*	*2 (2/2)*	*2 (2/2)*	*2 (0/3)*	***0 (0/2)*** *
Palpebral closure	0 (0/0)	0 (0/0)	0 (0/0)	0 (0/0)	0 (0/1)	0 (0/0)	0 (0/0)	0 (0/0)	0 (0/0)	0 (0/0)	0 (0/0)	0 (0/0)	0 (0/0)	0 (0/0)	0 (0/0)	0 (0/1)
*Ruffled fur*	*0 (0/0)*	*0 (0/0)*	*0 (0/0)*	***0 (0/1)*** *	*0 (0/0)*	*0 (0/0.12)*	*0 (0/0)*	*0 (0/0)*	*0 (0/0)*	0 (0/1)	*0 (0/0)*	*0 (0/0)*	*0 (0/0)*	*0 (0/1)*	*0 (0/0)*	***0 (0/1)*** *
*Skin color*	*1 (1/1)*	*1 (1/1)*	*1 (1/1)*	*1 (1/1)*	*1 (1/1)*	*1 (1/1)*	*1 (1/1)*	*1 (1/1)*	*1 (1/1)*	*1 (1/1)*	*1 (1/1)*	*1 (1/1)*	*1 (1/1)*	*1 (1/1)*	*1 (1/1)*	***0 (0/1)*** *
Heart rate	1 (1/1)	1 (1/1)	1 (1/1)	1 (1/1)	1 (1/1)	1 (1/1)	1 (1/1)	1 (1/1)	1 (1/1)	1 (1/1)	1 (1/1)	1 (1/1)	1 (1/1)	1 (1/1)	1 (1/1)	1 (0/1)
Tears	0 (0/0)	0 (0/0)	0 (0/0)	0 (0/0)	0 (0/0)	0 (0/0)	0 (0/0)	0 (0/0)	0 (0/0)	0 (0/0)	0 (0/0)	0 (0/0)	0 (0/0)	0 (0/0)	0 (0/0)	0 (0/0)
Salivation	0 (0/0)	0 (0/0)	0 (0/0)	0 (0/0)	0 (0/0)	0 (0/0)	0 (0/0)	0 (0/0)	0 (0/0)	0 (0/0)	0 (0/0)	0 (0/0)	0 (0/0)	0 (0/0)	0 (0/0)	0 (0/0)
**Muscle tone and strength (early CM parameter)**																
Grip strength	2 (2/2)	2 (2/2)	2 (2/2)	2 (2/2)	2 (2/2)	2 (2/2)	2 (2/2)	2 (2/2)	2 (2/2)	2 (2/2)	2 (2/2)	2 (2/2)	2 (2/2)	2 (2/2)	2 (2/2)	2 (2/2)
Body tone	1 (1/1)	1 (1/1)	1 (1/1)	1 (0/1)	1 (1/1)	1 (1/1)	1 (1/1)	1 (1/1)	1 (1/1)	1 (0/1)	1 (1/1)	1 (1/1)	1 (1/1)	1 (1/1)	1 (1/1)	1 (0/1)
Limb tone	1 (1/1)	1 (1/1)	1 (1/1)	1 (1/1)	1 (1/1)	1 (1/1)	1 (1/1)	1 (1/1)	1 (1/1)	1 (1/1)	1 (1/1)	1 (1/1)	1 (1/1)	1 (1/1)	1 (1/1)	1 (0/1)
Abdominal tone	1 (1/1)	1 (1/1)	1 (1/1)	1 (1/1)	1 (1/1)	1 (1/1)	1 (1/1)	1 (1/1)	1 (1/1)	1 (1/1)	1 (1/1)	1 (1/1)	1 (1/1)	1 (1/1)	1 (1/1)	1 (0/1)

Values of individual parameters of Cerebral Malaria (CM) at days 3 and 6 post-infection are shown. Animals infected with malarial parasite (PbA, Pch, PyNXL and PyXL) were compared to animals of the same background inoculated with uninfected RBC. Data are shown as median (upper/lower quartile) or mean ± SD when appropriated. N = 12–15/group.

*p<0.05 or less by Wilcoxon Signed Rank Test.

### Long-lasting cognitive impairment is present in animals that displayed initial neurologic signs of CM

**Figure 2:**
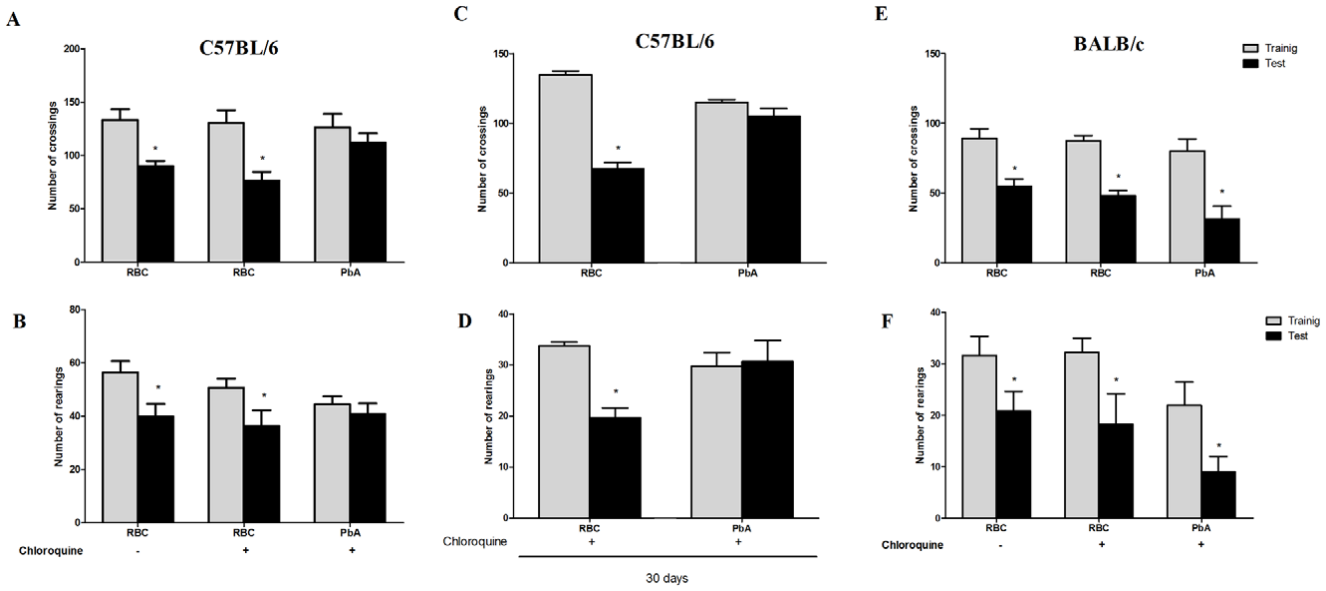
Open field task analysis identifies mouse strains that are susceptible to CM after PbA infection. C57BL/6 (A–D) and BALB/c (E and F) mice (n = 12–20/group) were infected with PbA (10^6^ PRBC i.p.) and treated with chloroquine (25 mg/kg) starting at day 6 post-infection. Data are expressed as mean ± S.E.M. of crossings (A, C, and E) and rearings (B, D and F) during training (*gray bars*) and test (*black bars*) sessions performed at days 15 and 16 (A,B,E, and F) or 30 and 31 (C and D) respectively; * significant difference between groups in training and test sessions (Student's T test, *p<0.05 A and B, E and F*; Mann Whitney test, *p<0.05 to C and D*).

To investigate the occurrence of late cognitive impairment, PbA-infected C57BL/6 mice that had early signs of CM as detected by the SHIRPA protocol were treated from day 6 to 12 with chloroquine and submitted to the open field-task analysis at day 15 post infection. Chloroquine treatment was very effective in controlling parasitemia, since infected red blood cell counts were reduced to 0.66±0.6% at day 16 and parasites were not recovered at day 30 (1.1±0.56%) post infection. There were no differences in the numbers of crossings and rearings observed when groups of PbA-infected C57Bl/6 and BALB/c mice subjected to the same rescue treatment with chloroquine were studied in the training session (Figure 2). In the test session, non-infected C57BL/6 mice treated with chloroquine or saline demonstrated a significant decrease in the numbers of crossings and rearings, indicating intact cognitive skills. In contrast, there was no reduction in crossings or rearings in PbA-infected C57BL/6 mice rescued with chloroquine (Figure 2A, B; right bars), indicating diminished cognitive capacity [20]. Importantly, the decrease in cognitive ability was persistent for at least 30 days indicating a long lasting dysfunction (Figure 2C, D). In parallel, PbA-infected BALB/c mice that did not have CM based on SHIRPA analysis (Table 1), but were, nonetheless, treated with chloroquine showed a significant reduction in both crossings and rearings (Figure 2E, F, p<0.05, Student's T Test) that was not different from what was observed in non-infected controls. Thus, despite being infected with PbA, as confirmed by parasitological examinations, BALB/c mice do not develop CM and its sequelae, i.e., late cognitive impairment.

**Figure 3:**
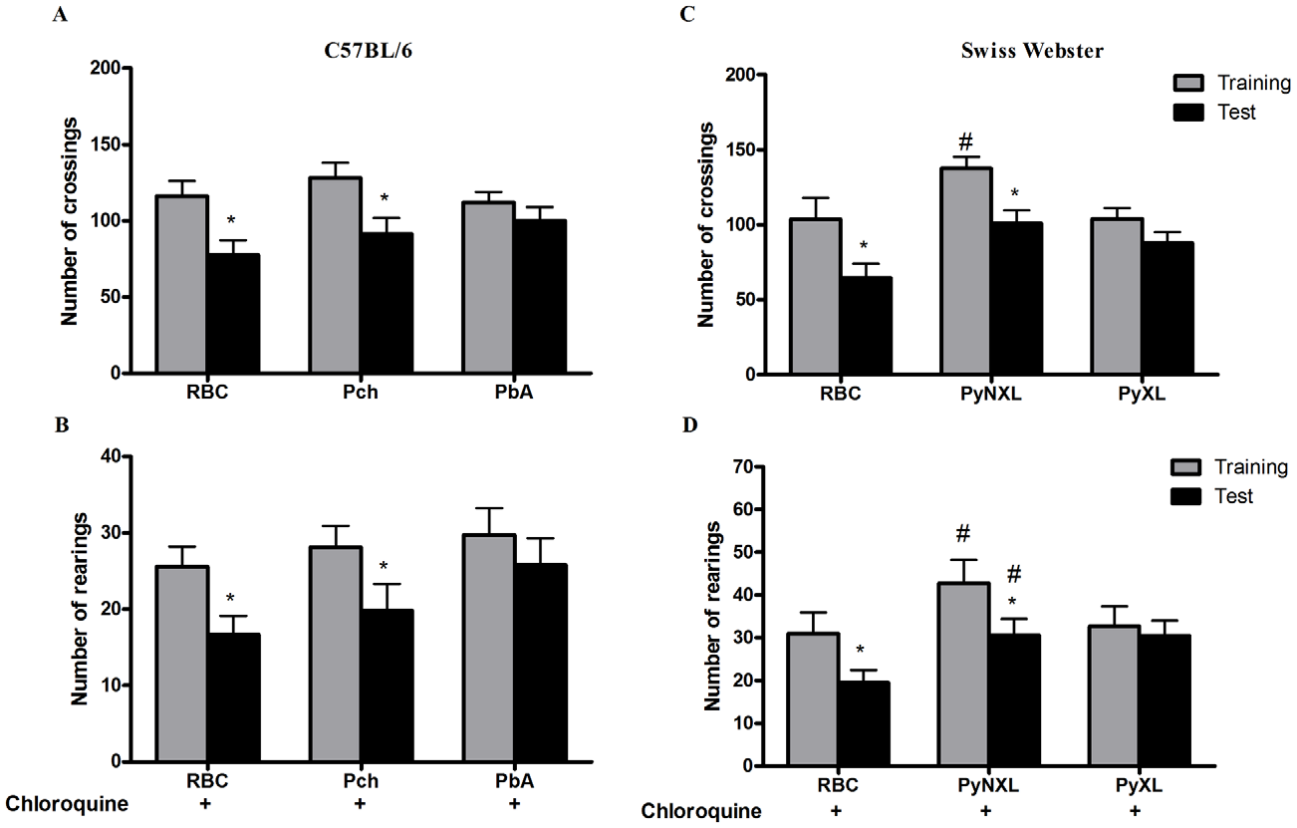
Impaired performance in the open field task is associated with development of CM. C57BL/6 mice (n = 12–20/group) were infected with PbA or Pch (10^6^ PRBC, A and B), or SW mice (n = 10–12/group) were infected with PyNXL or PyXL (10^6^ PRBC, C and D) and treated with chloroquine (25 mg/kg) starting at day 6 post-infection. Control groups were inoculated with the same number of uninfected RBC. Data are expressed as mean ± S.D. of crossings (*A and C*) and rearings (*B and D*) of training (*gray bars*) and test (*black bars*) sessions performed on days 15 and 16 post-infection respectively; *significant difference between groups in training and test studies (Student's T test, *p<0.05*).

Importantly, when C57BL6 mice were infected with Pch, a *Plasmodium* strain that does not induce CM [13] (Table 1), the pattern was similar to that of uninfected animals and CM-resistant BALB/c mice (Figure 3A, B). Therefore, even though C57BL/6 mice are susceptible to CM, when animals of this genetic background are infected with a *Plasmodium* strain that does not cause central nervous system involvement they do not develop signs of CM or consequent cognitive impairment based on our tools of detection. Conversely, cognitive impairment identified by our analytic instruments was not restricted to the C57BL/6 background since it was also observed in SW mice. SW mice infected with lethal strain PyXL [21] developed early signs of cerebral dysfunction that was not detected after infection with a non-lethal PyNXL strain (Table 1). SW mice infected with PyNXL showed a significant reduction in the numbers of test events when training and testing sessions were compared and the pattern was not different from non-infected control animals (Figure 3C, D). Nevertheless, when SW mice infected with PyXL were subjected to testing there was no reduction in test events in training and testing sessions (Figure 3C, D, right bars). A similar pattern was observed in PbA-infected C57BL/6 animals. Finally, we also performed experiments on PbA infected C57BL/6 animals that were depleted of CD8^+^ lymphocytes by treatment with anti-CD8 monoclonal antibody. CD8^+^ cells were previously shown to have an important role in CM [22]. In agreement with previous reports, single dose treatment with anti-CD8 temporarily reverse or stabilize the progression of CM [22], [23]. However, parasitemia and, consequently, anemia, are persistent in anti-CD8 treated mice and probably contribute to late deaths observed in these animals [22], [23]. In our hands, the first death in the anti-CD8 treated group was observed on day 13, but the majority of deaths occurred later on days 16–18. Importantly, the results from an open-field test can be altered if the mice are seriously ill, since the motor activity and general behavior are usually affected under this condition, interfering with the performance of the animals during the test. Therefore, to ensure that the results of the cognitive tests were not reflecting compromised behavior due to an ongoing severe systemic illness we decided to perform the experiments on animals that were treated both with chloroquine and anti-CD8. In fact, combined treatment with chloroquine and anti-CD8 monoclonal antibody prevented the occurrence of cognitive damage in these animals (reduction in crossings/rearings between training and testing sessions in untreated animals 34.0/32.5% versus reduction in crossings/rearings between training and testing sessions in anti-CD8 treated animals 13.0/0.0%). Together, these results indicate a clear correlation between the occurrence of CM and the development of late cognitive impairment.

### Different memory tasks are affected after CM

To determine if CM differentially influences memory skills, we submitted mice to different cognitive tasks including step-down latency and inhibitory avoidance, continuous multiple-trials step-down inhibitory avoidance and object recognition task. For this purpose, we elected to use PbA infection in C57BL/6 and BALB/c strain as positive and negative comparative models, respectively.

**Figure 4:**
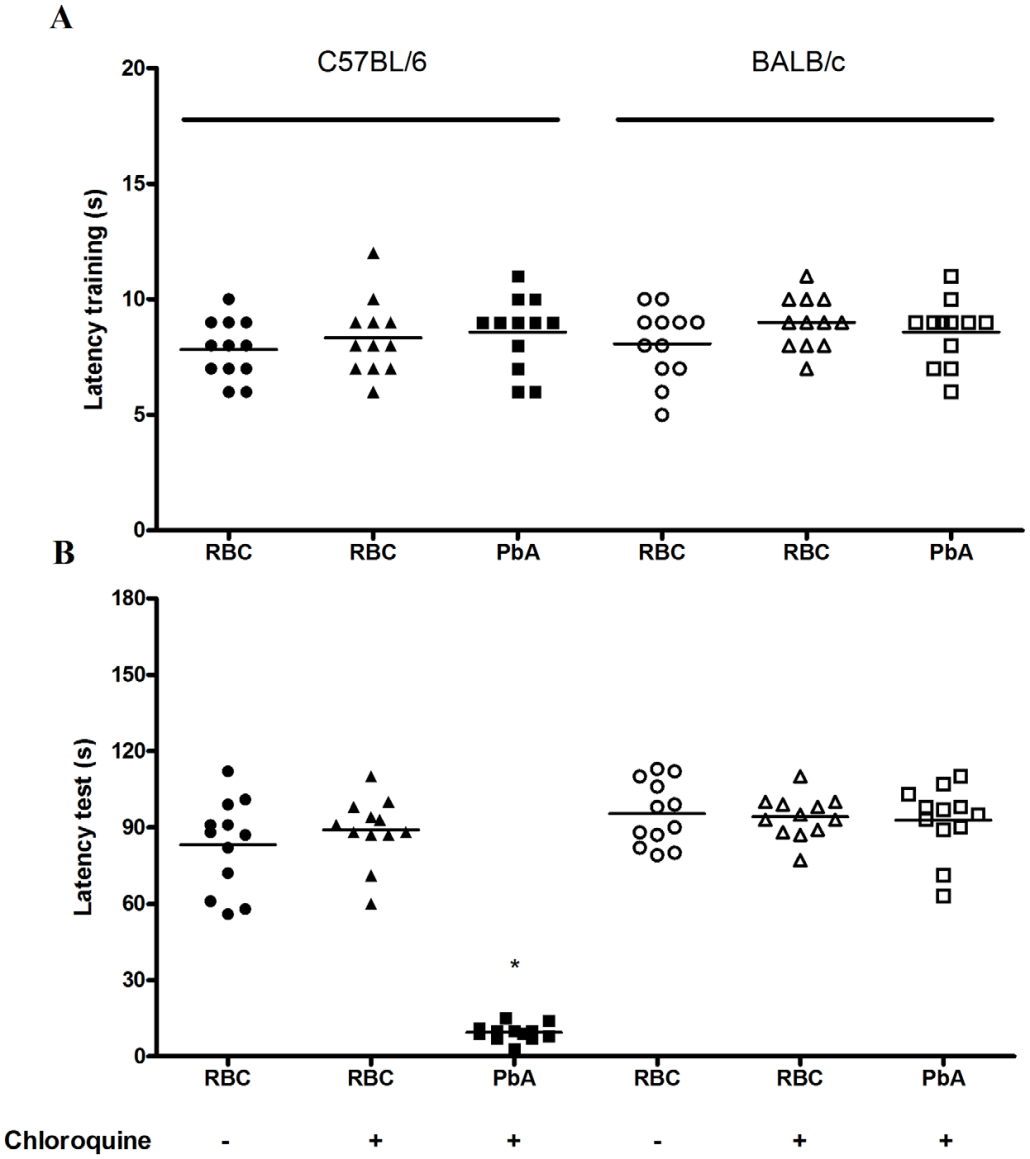
Aversive memory is also affected by CM as detected by the inhibitory avoidance task. C57BL/6 and BALB/c mice (n = 12–20/group) were infected with PbA (10^6^ PRBC) and treated with chloroquine starting at day 6 post-infection. Control groups were inoculated with the same number of uninfected RBC. (A) On day 15 all animals were subjected to a training session of inhibitory avoidance task, where the latency time on the platform is recorded and an electrical shock is given immediately after the mice step on the bars. (B) 24 h later, aversive memory was tested by recording the latency time on the platform (with a cut-off of 180 sec). Data are expressed as individual values and horizontal lines represent the median latencies, in seconds; *Significant difference compared with uninfected controls (comparisons among groups were performed by Mann-Whitney U test; individual groups were analyzed by Wilcoxon tests, *p<0.05*).

The step-down latency and inhibitory avoidance in the test session at day 15 post infection (Figure 4) was not different from training and test in PbA-infected C57BL6 mice treated with chloroquine (mean of latency of 9 and 9.5 s, training and test sessions respectively; Z = −1.075; p = 0.282, Wilcoxon's Test), suggesting impairment in aversive memory. On the contrary, non-infected mice treated with chloroquine or saline showed an increase in step-down latency, indicating intact aversive memory, when comparing their behavior in training and test sessions. A similar pattern was seen with Pb-infected BALB/c mice, where comparisons between infected and non-infected mice were not statistically different (Figure 4).

**Figure 5:**
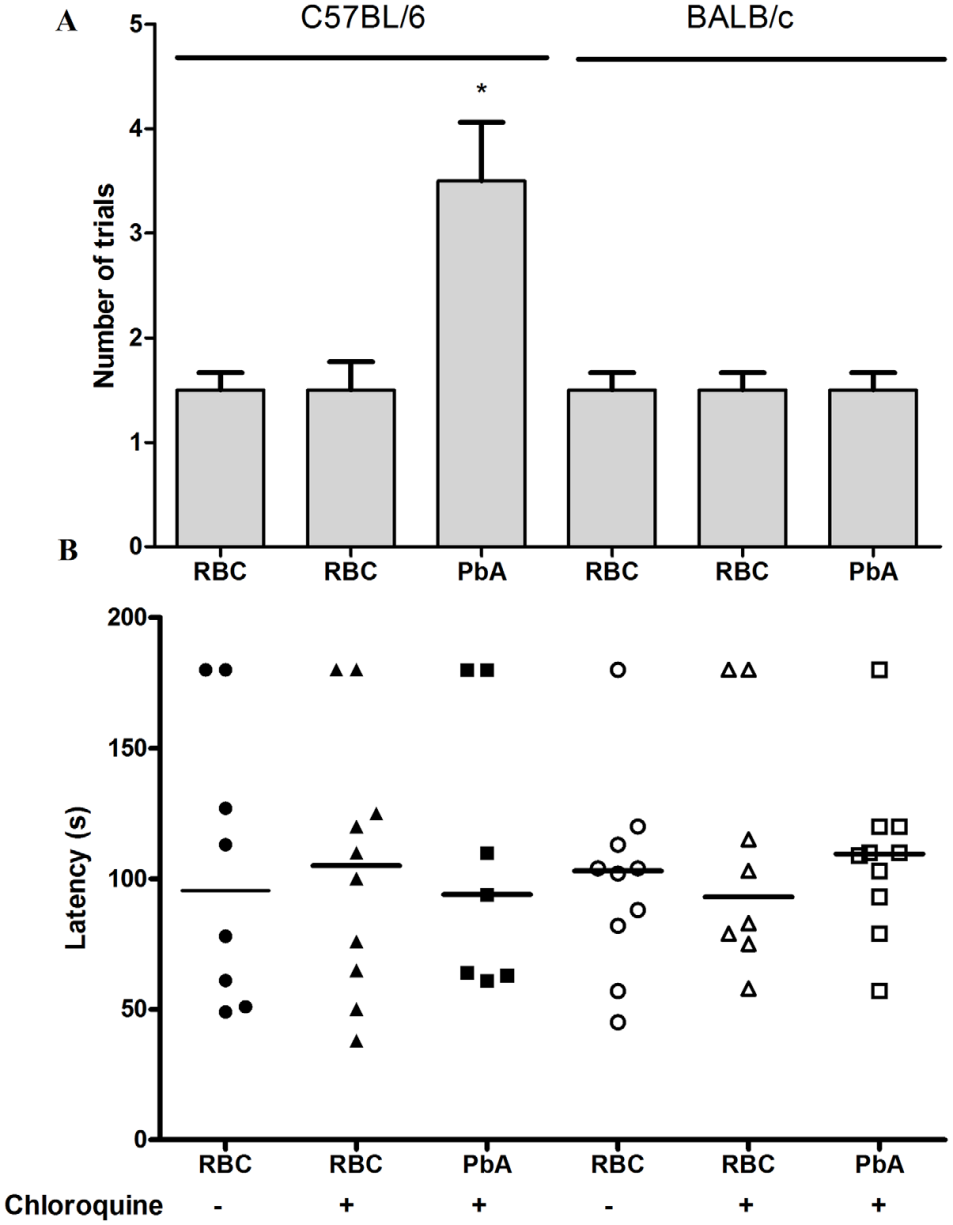
Long term aversive memory is not affected by CM as detected by continuous multiple-trials step-down inhibitory avoidance. C57BL/6 and BALB/c mice (n = 12–20/group) were infected with PbA (10^6^ PRBC) and treated with chloroquine starting at day 6 post-infection. Control groups were inoculated with the same number of uninfected RBC. On day 15 all the animals were submitted to a training session that consisted of a 0.3 mA 2.0 sec foot shock at the time that the animal stepped down on the grid. (A) Number of trials needed to achieve acquisition criterion (180 sec on the platform) one hour and thirty minutes after the training session. (B) Latency period on the platform 24 hours after the training session (cut-off at 180 seconds). Data are expressed as mean ± S.E.M. of the number of trials required to reach acquisition criterion (50 sec on the platform) in (A) and as individual values with median represented by a horizontal line in (B). *Significant difference between groups (comparisons among groups were performed by Mann-Whitney U test, the within individual groups were analyzed by Wilcoxon tests, *p<0.05*).

When we applied the continuous multiple trials step-down inhibitory avoidance task analysis (Figure 5), we observed a significant increase in the number of training trials required to reach the acquisition criterion (50 sec on the platform) with PbA-infected C57Bl/6 mice treated with chloroquine as compared to the non-infected controls (f_(5–54)_ = 8; p = 0.0001, Wilcoxon's Test). The results of this task suggest that PbA-infected C57Bl/6 mice required approximately two times more stimulation to reach the acquisition criterion compared to non-infected animals receiving the same treatment, indicating learning impairment after recovery from CM [24]. As expected, PbA-infected BALB/c mice did not show any differences in the number of training trials required to reach the acquisition criterion when compared to non-infected controls. In the retention test, there was no difference between groups at all the time points tested. Therefore, learning ability, but not long term aversive memory retention skills, is impaired in PbA-infected C57BL/6 mice.

**Figure 6:**
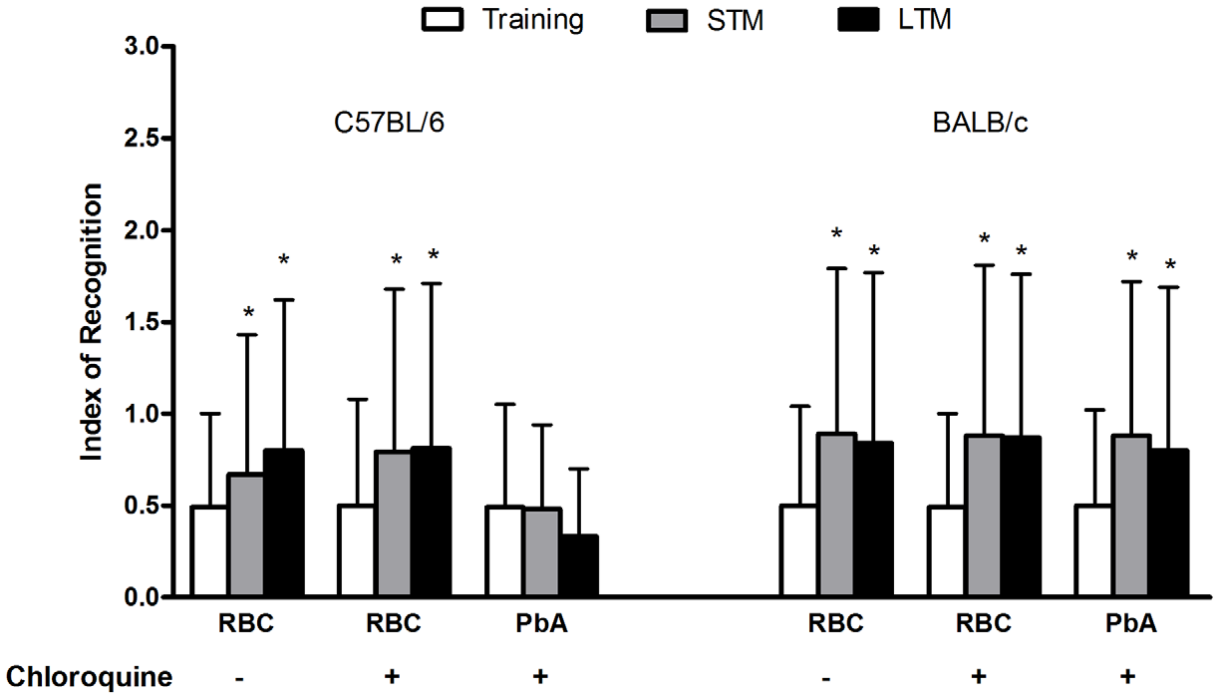
The ability to recognize new objects is impaired in mice after CM. C57BL/6 and BALB/c mice (n = 12–20/group) were infected with PbA (10^6^ PRBC) and treated with chloroquine starting at day 6 post-infection. Control groups were inoculated with the same number of uninfected RBC. On day 15 all the animals were submitted to memory recognition task. Results are shown as mean ± S.E.M. of the object recognition index calculated as described in Materials and Methods. *p<0.05 Kruskal–Wallis analyses of variance followed by Mann–Whitney U-tests.

PbA-infected C57Bl/6 mice treated with chloroquine showed an impairment of novel object recognition memory, i.e., they did not spend a significantly higher percentage of time exploring the novel object during short (Z = −1.782; p = 0.075, Kruskal-Wallis's Test) or long-term (Z = −1.753; p = 0.080, Kruskal-Wallis's Test) retention test sessions in comparison to the training trial (Figure 6). In contrast, this pattern was not reproduced in PbA-infected BALB/c mice (Figure 6). This result indicates that, as in other memory tasks, CM is associated with late deficits in cognition and memory skills that are not shared by infected animals that did not have clinical or neurobehavioural evidence for CM.

### CM is associated with increased oxidative stress in brain tissues

**Figure 7:**
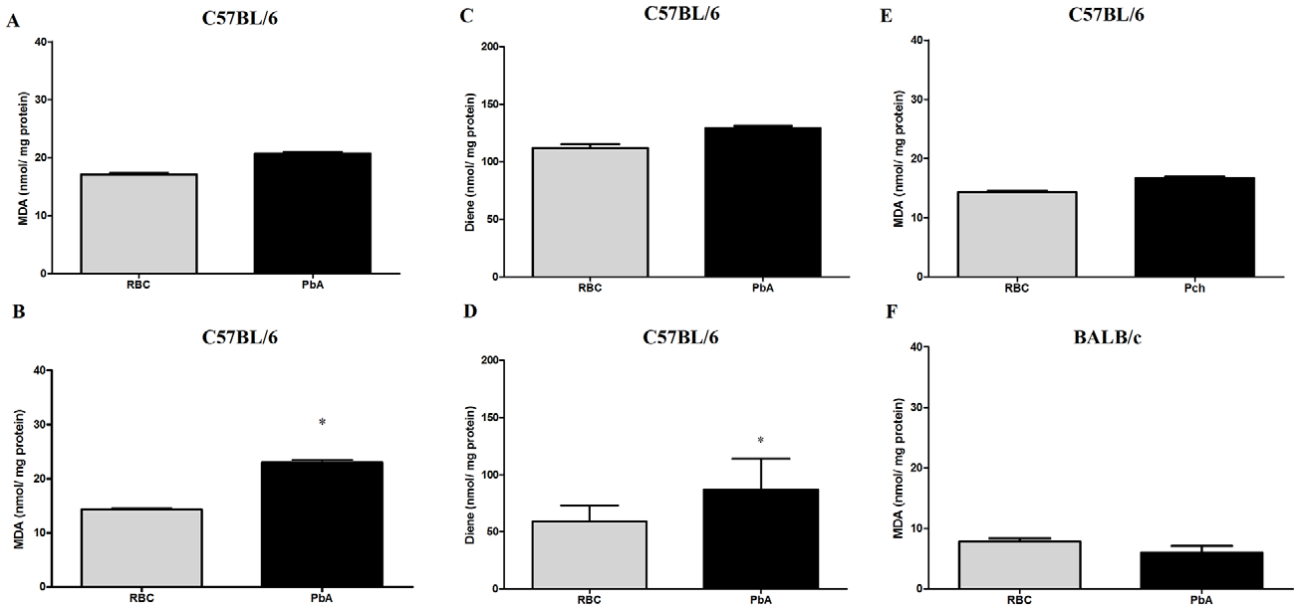
Oxidative stress is increased in the brains of mice with CM. Oxidative damage was assayed by measuring TBARS (A, B, E and F) and conjugated diene (C and D) formation in brains on days 3 (A and C) and 6 (B, D, E and F) post-infection of C57BL/6 with PbA or Pch (10^6^ PRBC, n = 10/group, A–E) or BALB/c with PbA (10^6^ PRBC, n = 6, F). Control groups received the same number of uninfected RBC (10^6^). Results are expressed as mean ± S.E.M. and * represents *p*< 0.05 compared to RBC group according to Student's t-test.

Oxidative stress is thought to be an important mechanism in the pathogenesis of neurodegenerative diseases and in sepsis-associated encephalopathy [25], [26]. To examine this issue in experimental CM, we measured lipid peroxidation by the production of MDA, and the formation of diene conjugated species. On day 3 post infection, no significant differences in lipid peroxidation were detected in brains of C57BL/6 mice infected with PbA compared to those inoculated with control RBC (Figure 7A, C). On day 6 post infection, however, the amount of both MDA and diene conjugates (Figure 7B, D, p<0.05, Student's T Test) were increased in brain tissue from PbA-infected mice when compared to the RBC group. Conversely, C57BL/6 mice infected with Pch and BALB/c mice infected with PbA, which do not develop CM, did not show increased production of MDA (Figure 7E, F) or diene conjugates (data not shown). These data identify oxidative stress in the brains of C57BL/6 mice infected with PbA but not in non-infected controls or mice infected with Pch, a *Plasmodium* strain that does not cause CM, suggesting that oxidative injury is a component of neurological impairment and, potentially, cognitive dysfunction in murine CM.

### Treatment with desferoxamine and *N*-acetylcysteine prevent late cognitive impairment after CM

**Figure 8:**
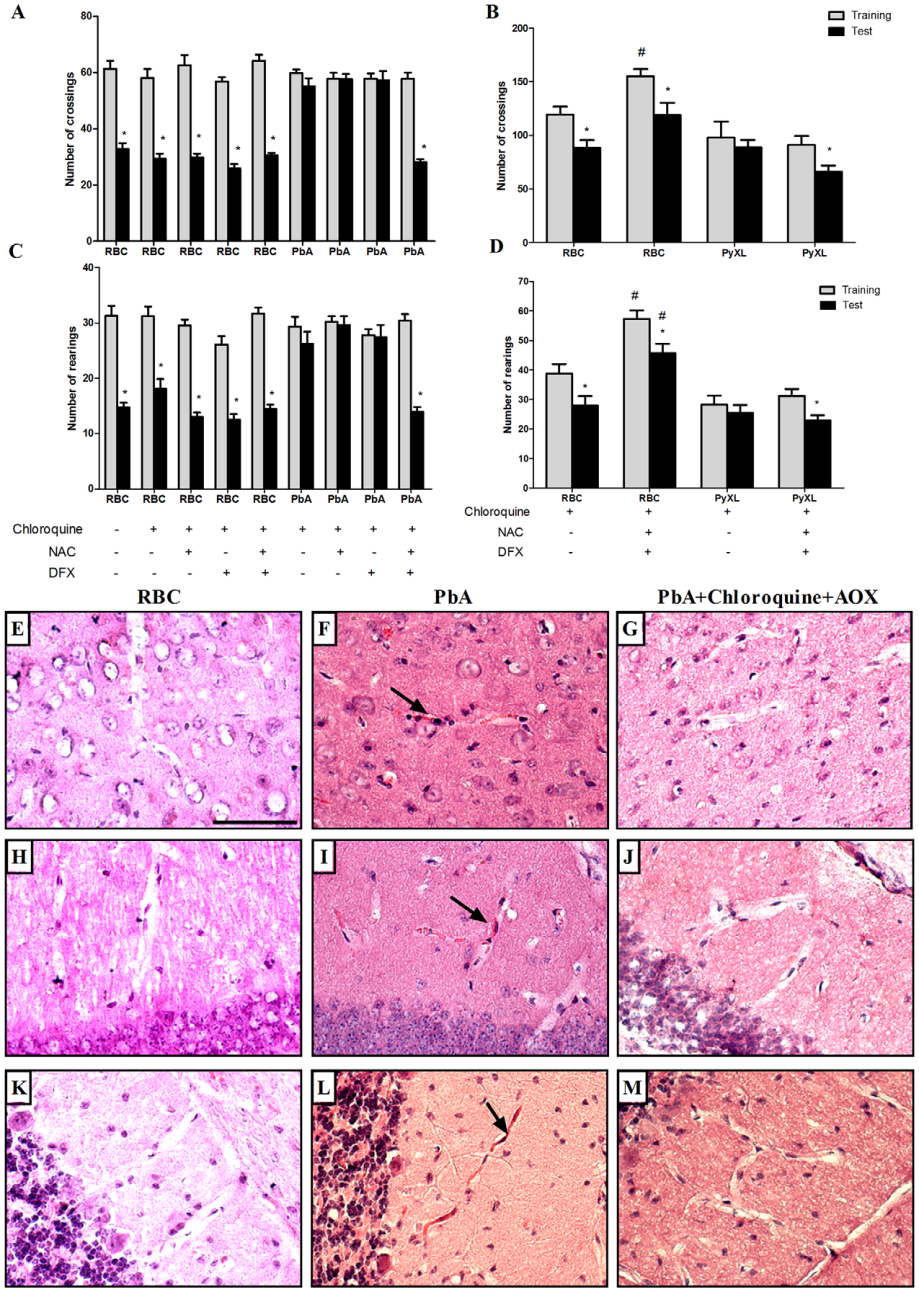
Additive antioxidant treatment prevents cognitive impairment after CM. C57BL/6 (A and B) and SW mice (C and D) (n = 12–20/group) were infected with PbA or PyXL, respectively (10^6^ PRBC). As a control, one group was inoculated with the same number of uninfected RBC (n = 6/group). Starting on day 6 post infection, infected mice were divided into 2 groups and treated orally with chloroquine (25 mg/kg b.w.), chloroquine plus desferoxamine (DFX, 20 mg/kg b.w., i.p.), chloroquine plus N-acetylcysteine (NAC, 20 mg/kg b.w., i.p.) or with the combination of chloroquine/DFX/NAC for 7 days. On day 15 and 16 post-infection all the animals were submitted to open field task training and test sessions, respectively. Data are expressed as mean ± S.E.M. of crossings (*A and C*) and rearings (*B and D*) in training (*gray bars*) and test (*black bars*) sessions; *significant difference between groups in training and test sessions (*p*<0.05, Student's T test). Panels E–M ilustrate histological examinations (H&E staining) of different brain regions in non-infected (RBC), infected (PRBC, PbA 10^6^) and mice treated with the combination of chloroquine/DFX/NAC (PbA+Cq+AOX). E–G) cerebral cortex; H–J) hippocampus; K–M) and cerebellum. Microvascular congestion and plugging (arrows) were detected in all analyzed regions in PbA-infected mice, but were not seen in controls or treated animals rescued with chloroquine and additive antioxidants. Scale bar, 100 µm.

Taoufiq and coworkers [27] proposed that the protection of the endothelium by antioxidant delivery may constitute a relevant strategy in CM. Therefore, we asked if antioxidants used as an additive together with antimalarials therapy would reduce subsequent cognitive impairment in mice that developed early clinical signs of CM. We treated PbA-infected C57BL/6 that showed signs of CM, detected by the SHIRPA protocol, with chloroquine plus a combination of desferoxamine and *N*-acetylcysteine treatment starting when antimalarial treatment was initiated on day 6 post-infection and continuing for 7 days. As described previously, treatment with chloroquine alone dramatically reduced mortality and parasitemia, but did not prevent cognitive damage (Figure 2). On the other hand, treatment with desferoxamine or *N*-acetylcysteine alone or in combination had no effect on the parasitemia curve (data not shown). We found that the treatment with a combination of chloroquine, desferoxamine and *N*-acetylcysteine ameliorated cognitive impairment in infected mice. Importantly, combination of chloroquine, desferoxamine and *N*-acetylcysteine was equally effective in controlling parasitemia as the treatment with chloroquine alone (0.66%±0.65 in chloroquine treated animals vs 0.71%±0.49 in animals with combination treatment, ns). Figure 8 shows that there was a significant reduction in numbers of crossing and rearing events when analysis in test and training sessions of mice treated with anti-parasitic and an antioxidant drugs (p<0.05) was compared to analysis of animals given chloroquine alone. The combined administration of desferoxamine and *N*-acetylcysteine is a necessary condition, since when chloroquine was given with either desferoxamine or *N*-acetylcysteine we did not see protection against the cognitive damage (Figure 8A, B). Combination therapy was also able to abolish microvascular congestion and plugging detected by histological examinations of the cortex, hippocampus and cerebellum of treated mice (Figure 8, panels G, J and M) at day 7 post-infection, histologic features that were present in untreated mice with clinical signs of CM (Figure 8, panels F,I and L). Administration of desferoxamine plus *N*-acetylcysteine without chloroquine did not protect animals from early death with high parasitemia and therefore could not be tested as a treatment for cognitive impairment. The protection of cognitive function by chloroquine together with desferoxamine and *N*-acetylcysteine was seen both in C57BL/6 mice infected with PbA (Figure 8A, B) and SW mice infected with PyXL and (Figure 8C, D), indicating that the additive therapy with antioxidants is able to prevent cognitive impairment due to CM in relevant models of the disease and diverse genetic backgrounds. Because artesunate has become the standard therapy to treat *P. falciparum* malaria in humans [28], we also performed an experiment in which the animals were treated with a combination of artesunate (100 mg/kg, b.w., p.o.) plus desferoxamine and *N*-acetylcysteine following the same protocol described above. As seen with chloroquine, combination therapy with artesunate was able to prevent the cognitive damage observed in untreated C57BL/6 mice infected with PbA (reduction in crossings/rearings between training and testing sessions in untreated animals 14.0/13.3% versus reduction in crossings/rearings between training and testing sessions in artesunate together with deferoxamine and *N*-acetylcysteine treated animals 32.8/23.8%).

## Discussion

More than 500,000 children develop CM in sub-Saharan Africa each year, of whom 110,000 die [29]. Additionally, survivors may not fully recover from CM since long-term cognitive impairment is observed in 12–14% of those individuals [6]. In a study conducted by Dugbartey and coworkers [7], children with a history of CM performed significantly poorer than those without previous CM in bimanual tactile discrimination, accuracy of visual scanning, visual memory, perceptual abstraction and rule learning skill, right ear auditory information processing, and dominant-hand motor speed. The social and economic burden of persistent cognitive dysfunction is not yet fully clear. Nevertheless, these residual deficits may affect future cognitive development in children, and this establishes the potential for devastating impact in adulthood. CM may thus be the chief cause of cognitive impairment in children in Sub-Saharan Africa and an important cause of cognitive impairment in adults in this region. Additional insights regarding the pathogenesis of cognitive deficits in CM and strategies for effective therapy to prevent this devastating complication are urgently required.

The natural history of cognitive dysfunction in experimental CM and its response to rescue therapy with antimalarial are unknown. Here we addressed these issues and provide new insights that may have clinical relevance. In the present work we demonstrated cognitive damage in animals rescued from CM by treatment with the antimalarial drugs chloroquine and artesunate in the early phase of the disease. In addition, we found that antioxidant agents that have previously been used in clinical regimens reduce cognitive dysfunction when given as additive to antimalarial therapy.

Experimental CM is characterized by brain edema, parenchymal lesions, blood brain barrier breakdown, and reduced cerebral blood flow. These pathophysiological responses are associated with impaired brain metabolism reflecting cellular injury and bioenergetic disturbances [30]. Magnetic resonance imaging studies suggest lesions in the corpus callosum and striatum [30]. The corpus callosum is one of the most prominent fiber systems of the mammalian brain. Patients with callosal damage cannot read text presented in the left visual field, and animals in which the callosum is divided, and sensory input restricted to one hemisphere, fail to show interhemispheric transfer of learning [31]. Taken together, these date suggest that damage in specific regions of the brain due to CM could generate cognitive damage as well as lack of memory or learning, similar to what was observed in neurocognitive impairment following CM in African children [4]. Additional studies are required to elucidate the mechanisms of central nervous system injury in children with CM as a necessary precursor to the development of interventions to prevent consequent long-term cognitive impairment [32]. We developed surrogate models that mimic clinical CM and its cognitive sequelae after parasitic cure by chloroquine, establishing invaluable tools to study mechanisms and consequences of cerebral involvement in malaria. We found that distinct cognitive abilities are affected in this condition, and that the use of antioxidant therapy concomitant with anti-malarial drugs was an effective therapy to prevent late cognitive damage to the host.

In experimental malaria, infection can vary in severity depending on the species and strain of *Plasmodium*, the dose of parasites and the mouse genetic background. We chose our innoculum based on previous work on experimental CM in the literature [12], [19], [33], [34], [35], but we recognize the possibility that different results could have been obtained if we had used a mild infection model. In non-lethal infections, such as those caused by *Pch and Py* 17XNL, resolution generally results in immunity to a second challenge with the same strain, but not to a heterologous parasite. Some parasite strains are lethal only to a particular strain of mice (for instance *Pch* to 129sv, A/J and DBA/2 mice) and some are uniformly lethal (*P. berghei* ANKA, *Py* 17XL or YM), indicating that parasite associated factors as well as the host genetic background interact to determine lethality [13]. In the PbA model, the genetic background of the murine host is extremely important and modulates the disease outcome. For instance, the Th-1 biased C57BL/6 mouse is susceptible to the development of CM, whereas the Th-2 biased BALB/c mouse is resistant [12]. Although PbA infection is regarded as a standard model of experimental CM, there have been conflicting results using the Py 17XL parasite as a CM model. Contrary to PbA, Py 17XL has been described to induce high parasitemia, massive anemia and kidney failure without CM (for review see Engwerda et al., [11]). On the other hand, other studies report that Py 17XL induces clear signs of CM and is a useful model of this condition in the laboratory setting [13], [21], [36]. We detected high parasitemia (32%) at day 7 after Py 17XL infection and these animals exhibited signs of cerebral dysfunction when submitted to the SIRPA protocol. Because we were able to establish sensitive and reproducible methods by which CM could be unequivocally demonstrated by performing tests described in the SHIRPA protocol [19], [37], our findings are consistent with previous literature indication that Py 17XL induces important dysfunctions in the central nervous system.

Based on previous studies [19], C57BL/6 mice with CM develop a wide range of behavioral and functional alterations as the syndrome progresses, and significant impairment in all functional categories when assessed 36 hours prior to death. Reflex, sensory function and neuropsychiatric state are altered in the early phase of malaria infection, and muscle tone, strength and autonomic functions are affected in animals with CM exclusively. We confirmed these findings in several models of CM. We also observed that C57BL/6 mice treated with chloroquine are rescued to basal locomotor activity when tested by the SHIRPA protocol (data not shown). Nevertheless, a cognitive deficit persists and was clearly demonstrated when the animals were subjected to specific tasks, such as the memory habituation open-field test performed 15 and 30 days after CM, indicating that cure of the parasitic infection does not prevent the development of late cognitive sequelae once CM is established. Furthermore, C57BL/6 mice infected with Pch developed clinical signs of infection but failed to develop CM and cognitive damage, indicating that cognitive impairment is not an unavoidable consequence of systemic malarial infection in C57BL/6 mice, but rather is associated with the development of clinically detected CM. We also found that severe infection without clinically established CM is not sufficient to trigger cognitive impairment using the PbA-infected BALB/c mice model. Taken together, these data document a strict correlation between development of CM and long-lasting cognitive impairment in surrogate models of malaria. It is our working hypothesis that acute cerebral malaria that leads to death in the absence of rescue therapy and long term cognitive dysfunction in animals that have been rescued with chloroquine share some of the same cellular and molecular mechanisms, and that the substrate for long term cognitive dysfunction is initiated by cerebral injury during the acute period of untreated cerebral malaria. We do not exclude, however, the possibility that long term cognitive dysfunction may also have complex mechanisms that are independent of those that cause neurologic injury and death during acute cerebral malaria, and that these undefined mechanisms only operate if the animal survives. Future investigations are aimed to clarify this point.

Memory function is vulnerable to a variety of pathological process including neurodegenerative diseases, strokes, tumors, head trauma, hypoxia, cardiac surgery, malnutrition, attention-deficit disorder, depression, anxiety, medications, and normal aging [38]. One of the most elementary nonassociative learning tasks is that of behavioral habituation to a novel environment [39]. We identified deficits in memory habituation in open-field test analysis, which revealed long-term memory defect in mice with experimentally-induced CM. This deficit was unrelated to changes in basic exploratory or motor processes. Rather, it is likely to be directly related to impaired hippocampus-dependent memory processes [40], [41]. Additional target areas such as prefrontal cortex could also be involved, since reduced density of neuronal cells in this area is known to lead to orientation disturbances and memory problems in complex tasks [42], [43].

Memory habituation impairment was not the only late consequence of CM in our models as, in fact, several other cognitive deficits were documented in PbA-infected C57BL/6 mice. Step-down inhibitory avoidance learning triggers biochemical events in the hippocampus that are necessary for the retention of this task. The events are similar in many ways to those described for different types of long-term potentiation and other forms of neural plasticity [44], [45]. They are triggered by glutamate receptor activation and involve at least four different cascades led by different protein kinases (PK), including protein kinase G, PKC, calcium-calmodulin-dependent protein kinase II (CaMKII), and PKA. Several steps in these cascades have been implicated in other forms of learning that also involve the hippocampus (reviewed by Izquierdo & Medina [45]).

Step-down inhibitory avoidance involves learning, acquired generally in one single trial, and long-term aversive memory retention. C57BL/6-infected mice lack long-term memory retention ability (24 hours post-stimulus) (Figure 4B) and have deficits in learning even when they are submitted to multiple trials (Figure 5A). The inhibitory avoidance task relies heavily on the dorsal hippocampus, but also depends on the entorhinal and parietal cortex and is modulated by the amygdala [44], [45]. CM may, therefore, be affecting distinct areas in the brain to interrupt different facets of memory and task performing ability.

We found that object recognition is also impaired after CM. This task, originally developed by Ennaceur and Delacour [46], is based on the tendency of rodents to explore a novel object more than a familiar one. Because no rewarding or aversive stimulation is used during training, the learning occurs under conditions of relatively low stress or arousal [46]. We observed that PbA-infected C57BL/6 mice rescued from CM with chloroquine had significant impairment in novel object recognition memory compared with sham-infected mice. These findings are important since the novel object recognition task in rodents is a nonspatial, nonaversive memory test, in contrast to other tests performed in this study (habituation and aversive memories) [47]. Recognition of objects is thought to be a critical component of human declarative memory that is mainly dependent on the hippocampus. Object recognition is commonly impaired in human patients affected by neurodegenerative diseases, or who have suffered brain injury [47], [48].

We do not know if the cognitive defects are reversible, but our experiments indicate that they persist for at least 30 days after rescue from CM with chloroquine alone. Experiments are in progress to determine the duration of CM induced cognitive deficiency imposed by CM in these models.

The mechanisms for cognitive impairment in CM are not completely characterized, but inflammation and vascular dysfunction appear to be the basis of cerebral involvement [9]. During malarial infection, the host and parasites are under severe oxidative stress with increased production of reactive oxygen species (ROS) and NO by activated cells in the host [14]. When produced in large amounts, ROS and nitrogen intermediates may cause damage to the host tissue including the vascular endothelium. Endothelial damage may lead to increased vascular permeability and leukocyte and platelet adherence, all seen in cerebral malaria [49]. Despite being generally accepted, this view has been challenged by observations showing that gp91phox deficient mice and inhibitors of iNOS fail to modify the development of cerebral malaria in appropriate murine models [50], [51]. We have performed preliminary experiments using NOS deficient mice and observed that those animals, despite being susceptible to high parasitemia and early death with CM symptoms, were protected from the cognitive damage if treated with chloroquine at day 6 post infection. Together, these observations may suggest that the pathology leading to mortality during CM may occur via different mechanisms than that leading to cognitive dysfunction after successful rescue therapy. In this pathophysiologic milieu, antioxidants may be an effective strategy to counteract damage in CM, and metal chelators may be of particular interest [52].

Products of lipid peroxidation are markers for oxidative stress in several diseases and experimental models [53]. To characterize the oxidative damage during early events of CM we measured TBARS and conjugated diene formation on days 3 and 6 post infection. Our findings indicate a significant increase in oxidative stress in the brains of PbA-infected mice on day 6 post-infection, further suggesting antioxidants as a potential additive therapy to reduce cerebral damage and cognitive dysfunction during CM. Oxidative stress is associated with the development of neurodegenerative diseases and is important to the development of multiple organ dysfunction syndromes during sepsis [25], [26], providing a precedent for this approaches. In fact, combined antioxidant therapy with *N*-acetylcysteine and desferoxamine improves survival in sepsis induced by cecal ligation and puncture (CLP) in rats by decreasing oxidative stress and limiting mitochondrial dysfunction [54]. Barichello and coworkers [55] showed that the combined therapy also prevents late memory impairment in experimental sepsis. *N*-acetylcysteine is a well-known thiolic antioxidant that acts as a precursor for glutathione synthesis [56]. The reducing thiol group in *N*-acetylcysteine also reacts directly with ROS, leading to cellular protection against oxidative damage *in vitro* and *in vivo* [57]. Desferoxamine is a powerful iron chelator that can inhibit iron dependent free radical reactions and has been shown to diminish oxidant damage in several animal model of human disease [58], [59]. Previous studies have demonstrated that desferoxamine protects against brain ischemic injury in neonatal rats when administered after an ischemia-reperfusion insults [60]. In adult rats, desferoxamine protects against focal cerebral ischemia when given as a preconditioning stimulus 72 h before the ischemic insult [61]. In agreement with the protective effect of antioxidants in sepsis-induced brain dysfunction, we found that combined treatment with *N*-acetylcysteine, desferoxamine and chloroquine in PbA-infected C57BL/6 mice or Swiss mice infected with PyXL prevented cognitive damage as detected by the open-field task test, further indicating a role for oxidative stress in the development of cognitive dysfunction in experimental CM and providing an approach to modify this consequence of cerebral injury. In addition, our initial experiments indicate that antioxidants are effective as additive treatment in combination with artesunate as well. Because *N*-acethylcysteine and desferoxamine have been used in clinical treatment of human subjects and their pharmacologic profile and side effects are known, we suggest that these drugs should be examined as additive therapy for antimalarial drugs in clinical trials to investigate their potential to decrease, or prevent, cognitive damage after CM.

## Methods

### Animals

6–8 weeks old C57BL/6, BALB/c (n = 10/group per experiment) and *Swiss webster* (SW, n = 8/group) mice from the Oswaldo Cruz Foundation breeding unit, weighing 20 to 25 g, were used for the studies. The animals were kept at constant temperature (25°C) with free access to chow and water in a room with a 12 hour light/dark cycle. The experiments in these studies were approved by the Animal Welfare Committee of the Oswaldo Cruz Foundation under license number L033/09 (CEUA/FIOCRUZ). The guidelines followed by this Committee were created by the same institution that provided ethical approval.

### Drugs

N-acetylcysteine (Zambom Group S.p.A., Italy), desferoxamine (Novartis Bioscience S.A., Brazil), and chloroquine (Farmanguinhos, Oswaldo Cruz Foundation, Brazil) were directly dissolved in saline solution (NaCl 0.9%, w/v). The solutions were prepared immediately before use and were protected from the light before administration to the animals.

### Parasites, infection and disease assessment

Uncloned parasite lines of *Plasmodium berghei* ANKA, *Plasmodium chabaudi chabaudi* and *Plasmodium yoelii* were used in this study. *Plasmodium berghei* ANKA (PbA) parasitized red blood cells (PRBC) from BALB/c or C57BL/6 mice, *Plasmodium chabaudi chabaudi* (Pch) in C57BL/6 PRBC, *Plasmodium yoelii* non-lethal (PyNXL), and *Plasmodium yoelii* lethal (PyXL) in Swiss Webster PRBC donor strains were kept in liquid nitrogen and were thawed and passed into normal mice that served afterwards as parasite donors. 6–8 weeks old C57BL/6, BALB/c and *Swiss webster* (SW) mice were inoculated intraperitoneally with 0.2 mL suspension of 10^6^ parasitized red blood cells (n = 8–10/group). As a control group for infection, mice were inoculated with 10^6^ non parasitized red blood cells (RBC). Parasitaemia on days 3, 5, 7 and 10 and survival rate were recorded.

### Histopathology

On day 7 post-infection, animals of different groups (control, PbA-infected, and PbA-infected rescued with chloroquine and antioxidant; n = 3 per group) were transcardially perfused with 0.9% saline solution and 4% paraformaldehyde in PBS. The brains were carefully dissected, cryoprotected in 10%, 20%, and 30% sucrose at 4°C, and embedded in O.C.T. (Tissue-Tek) for frozen sectioning on a cryotome (Leica Microsystems). Parasagittal sections were cut at 12 µm and placed on slides for staining with haematoxylin and eosin (H&E – VETEC, Rio de Janeiro) for histological analysis by a blinded expert pathology. Sections were examined on an Axioplan light microscope (Zeiss, Germany).

### Experimental design

Mice were infected as described above. On day 3 and 6, they were subjected to SHIRPA protocol testing (see below) to identify neurobehavioral signs of CM. Animals that were positive for clinical signs of CM detected in this fashion were immediately started on chloroquine treatment (25 mg/kg b.w., orally) and were treated daily for 7 days (15 days analisis) or 21 days (30 days analysis). At day 15 post infection, the animals were subjected to a battery of behavioral tests to access cognitive function. As a control, uninfected mice received saline (when indicated) or chloroquine. An additional group of animals received additive antioxidant therapy with desferoxamine and N-acethylcisteine, (each 20 mg/kg b.w., intraperitoneally) from day 6 to 12 post infection, concomitant with chloroquine, and then were subjected to behavioral tasks on day 15.

In an additional experiment, mice were infected with 10^6^ PbA-PRBC. At day 6 mice were intraperiotoneally treated with a single dose of 0.5 mg anti-CD8 Mab obtained from Hybridomas 53-6.7 [62] and orally treated with chloroquine (25 mg/kg b.w.) during 7 days.

### Behavioral analysis by SHIRPA protocol

The behavioral testing was performed according to the SHIRPA protocol [63], 1997). The primary screen was performed as described for detection of CM by Lackner and coworkers [19]. Individual tests are described in Table 1.

### Open field task

Habituation to an open-field was carried out as described by Vianna and coworkers [39]. Animals were gently placed on an open field apparatus and allowed to explore the arena for 5 minutes (training session). 24 h later they were submitted to a similar open-field session (test session). Crossing of the black lines and rearing performed in both sessions were counted.

### Step-down inhibitory avoidance test

The step-down inhibitory avoidance test was performed as described by Quevedo et al., [64]. In the training trial, animals were placed on the platform and their latency to step down on the grid with all four paws was measured with an automatic device. Immediately after stepping down on the grid, the animals received a 0.4 mA, 2.0 seconds foot shock. A retention test trial was performed 24 h after training and permanence on the grid is recorded.

### Multiple-trials step-down inhibitory avoidance task

Continuous multiple-trials step-down inhibitory avoidance task testing was performed in the same step-down inhibitory avoidance apparatus, however, in the training session, animals were placed on the platform and immediately after stepping down on the grid, received a 0.3 mA, 2.0 seconds foot shock. 1 h 30 min later, this procedure was repeated until the mice remained on the platform for 50 seconds and the number of training trials required was recorded. On the following day the retention test was performed and the result was given by latency period on the platform, with a cut-off at 180 seconds [65], [66].

### Object recognition task

The object recognition task was carried out as described in previous studies [67]. Briefly, animals had the opportunity to explore the open field for 5 min. On the following day, a training session was conducted by placing individual mice for 5 min into the field in the center of the arena, in which two identical objects (object A1 and A2; Double Lego Toys) were positioned in two adjacent corners at 10 cm from the walls. In a short-term memory (STM) test (1.5 h after training), the mice explored the open field for 5 min in the presence of one familiar (A) and one novel (B) object. In a long-term memory (LTM) test (24 h after training), the mice explored the field for 5 min in the presence of the familiar (A) and different novel (C) object. Objects had only distinction in shape. The exploratory preference was defined as percentage of the total exploration time animal spent investigating the object A or the novel object and calculated for each animal by the ratio *T*_B or C_/(*T*_A_+*T*_B or C_) [*T*_A_ = time spent exploring the familiar object A; *T*_B or C_ = time spent exploring the novels objects B or C).

### Assessment of oxidative stress

To characterize the oxidative stress in murine brains, lipid peroxidation levels were measured by assays of thiobarbituric acid reactive species - TBARS [68] - and the formation of diene-conjugated species [69]. Brains from animals dying of CM were homogenized in cold phosphate buffer, pH 7.4 with BHT (final concentration 0.2%). Briefly, the samples (0.5 mL) were mixed with equal volume of thiobarbituric acid 0.67% (Sigma Chemical, St. Louis, MO) and then heated at 96°C for 30 min. TBARS were determined by the absorbance at 535 nm. To analyze diene-conjugate formation, lipids were extracted by partition on chloroform∶methanol (2∶1, v∶v) and the organic phase was submitted to espectrophotometric analysis at 234 nm. Results were expressed as malondialdehyde (MDA, ε = 1,56×10^5^M^−1^cm^−1^) and diene equivalents (ε = 2,95×10^4^M^−1^cm^−1^) per milligram of protein (BCA assay) [68].

### Statistical analysis

Data were expressed as mean ± SEM. Statistical significance of survival curves were evaluated by Log-rank (Mantel-Cox) and Gehan-Breslow-Wilcoxon tests. Statistical analysis from SHIRPA was performed by nonparametric test (Wilcoxon rank-sum test). Data from the open-field task were analyzed with ANOVA followed by Tukey post hoc and Student's T tests and expressed as mean ± SEM. Data from the inhibitory avoidance task, object recognition task and the number of training trials from continuous multiple trials step-down inhibitory avoidance are reported as median and interquartile ranges and comparisons among groups were performed using Mann–Whitney U tests. The variations within individual groups were analyzed by Wilcoxon's test. Difference in amounts of MDA and diene-conjugates were evaluated by Student's T test. In all comparisons, p<0.05 or less was taken as statistical significance.
